# Evaluation of Online Plan Adaptation Strategies for the 1.5T MR-linac Based on “First-In-Man” Treatments

**DOI:** 10.7759/cureus.2431

**Published:** 2018-04-05

**Authors:** Dennis Winkel, Gijsbert H Bol, Ilse H Kiekebosch, Bram Van Asselen, Petra S Kroon, Ina M Jürgenliemk-Schulz, Bas W Raaymakers

**Affiliations:** 1 Department of Radiotherapy, UMC Utrecht

**Keywords:** mr-linac, adaptive planning, adaptive radiotherapy, online replanning, lumbar spine bone metastases, mr-guided-rt, mr-igrt, mr guided radiotherapy

## Abstract

The superior soft tissue contrast provided by magnetic resonance (MR) images on the 1.5T MR-linac allows for the incorporation of patient anatomy information. In this retrospective case study, we present the simulated dosimetric effects and timings of full online replanning as compared to the five plan adaptation methods currently available on the 1.5T MR-linac treatment system. For this case, it is possible to create treatment plans with all six methods within a time slot suitable for an online treatment procedure. However, large dosimetric differences between the plan adaptation methods and full online replanning are present with regards to target coverage and dose to organs at risk (OARs).

## Introduction

Image-guided radiation therapy (IGRT) has become the standard in modern radiotherapy. Imaging prior to treatment can help reduce the effect of setup errors and the geometrical variations of the target volume and organs at risk (OARs) [1]. Most modern radiotherapy treatment systems are equipped with cone-beam computed tomography (CBCT), allowing the visualization of the tumor and nearby structures. This technique has greatly contributed towards precision therapy for target volumes that are clearly visible on CBCT. However, it yields relatively poor soft tissue contrast. Magnetic resonance imaging (MRI)-guided radiotherapy treatment systems are commercially available and used in clinical practice [2-3]. The superior soft tissue contrast provided by MR images on the 1.5T MR-linac allows for the incorporation of more detailed patient anatomy information. Diagnostic-quality MR images are available of the actual patient anatomy just before and during treatment, which are then used in an MR-guided online adaptive workflow. The 1.5T MR-linac system currently has five methods for plan adaptation. To our knowledge, these have not previously been evaluated on clinical data acquired during actual treatment on the MR-linac. In this retrospective case report, we present the dosimetric effects and timings of the five available plan adaptation options currently provided on the 1.5T MR-linac treatment system as well as full online replanning, in which a completely new plan is generated on the daily MRI with the actual patient anatomy.

## Case presentation

Patient data

For this dosimetric case study, we used data from one patient who participated in the first clinical study ever performed on the 1.5T MR-linac (FIM) [4]. In this trial, four patients with painful lumbar spine bone metastases were treated with a single fraction of 8Gy prescribed to the planning target volume, defined by the entire vertebral body, following certain dose criteria (Table 1).

**Table 1:** Clinically used dose criteria for MR-linac treatment of lumbar spine bone metastases for planning and plan evaluation.

Structure	Constraint
Planning Target Volume (PTV)	V_7.2Gy _> 90%,
	D_98%_ > 6.4Gy
	D_0.1cc_ < 8.8Gy
Spinal Cord	D_0.1cc _< 8.6Gy
Body	D_0.1cc _< 8.8Gy

**Figure 1:**
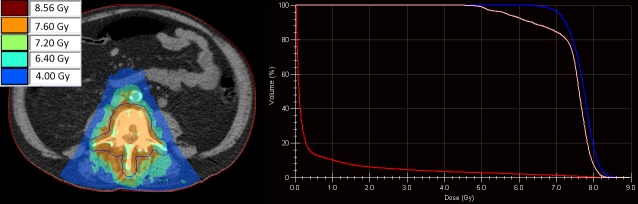
Dose distribution of the pre-treatment plan generated on the pre-treatment computed tomography (CT, left) with the corresponding dose volume histogram (right). Visible are the planning target volume (PTV, blue), spinal cord (pink), and body (red).

For each patient, a pre-treatment plan was available based on pre-treatment CT data (Figure 1). During the FIM treatment, an online MRI (three-dimensional spoiled gradient recalled acquisition in steady state (3D SPGR) T1-weighted (T1W), flip angle = 13˚, echo time (TE) = 3.6ms, repetition time (TR) = 5.7ms) was acquired. The online MRI was registered with the pre-treatment data to acquire the target and organ at risk contours and Hounsfield values using the deformable registration software ADMIRE (version. 1.13.3, Elekta AB, Stockholm, Sweden). For this case report, we randomly selected the dataset of a 65-year-old patient with bone metastases in lumbar vertebra 4 (L4) to explore the plan adaptation options. The clinical pre-treatment plan was used as the starting point.

Plan adaptation/approximation

In this retrospective case study, we simulated the dosimetric effects and timings of full online replanning and the five plan adaptation/approximation methods currently available on the 1.5T MR-linac treatment system using the Monaco (Version 5.40.00 build 1073, Elekta AB, Sweden) treatment planning software (TPS):

1.       Original Segments: Keep original segment locations and only modify isocenter.

2.      Adapt Segments: Reposition segments based on the isocenter shift.

3.      Optimize Weights: Optimize weights after adapting segments.

4.      Optimize Shapes: Optimize shapes and weights after adapting segments.

5.      Optimize -> Fluence Optimization: Remove segments and optimize towards original fluence.

**Figure 2:**
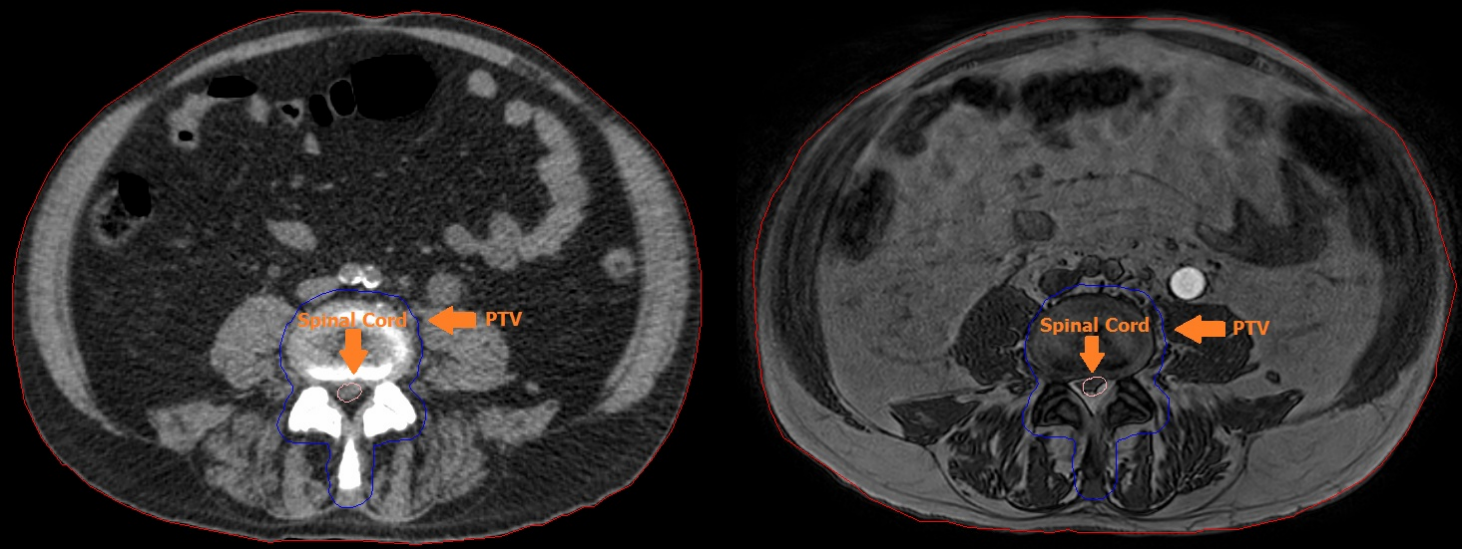
Pre-treatment Computed Tomography (CT, left) and online, just before treatment, Magnetic Resonance Image (MRI, right). Visible are the Planning Target Volume (PTV, blue), spinal cord (pink) and body (red). Small variations between the delineations can be observed.

Plan adaptation was performed based on the pre-treatment data and the daily MRI data (Figure 2). The plan quality was evaluated based on the clinical dose criteria. The time needed for plan adaptation or full online replanning toward the clinical dose criteria was measured.

Dosimetric results

**Figure 3:**
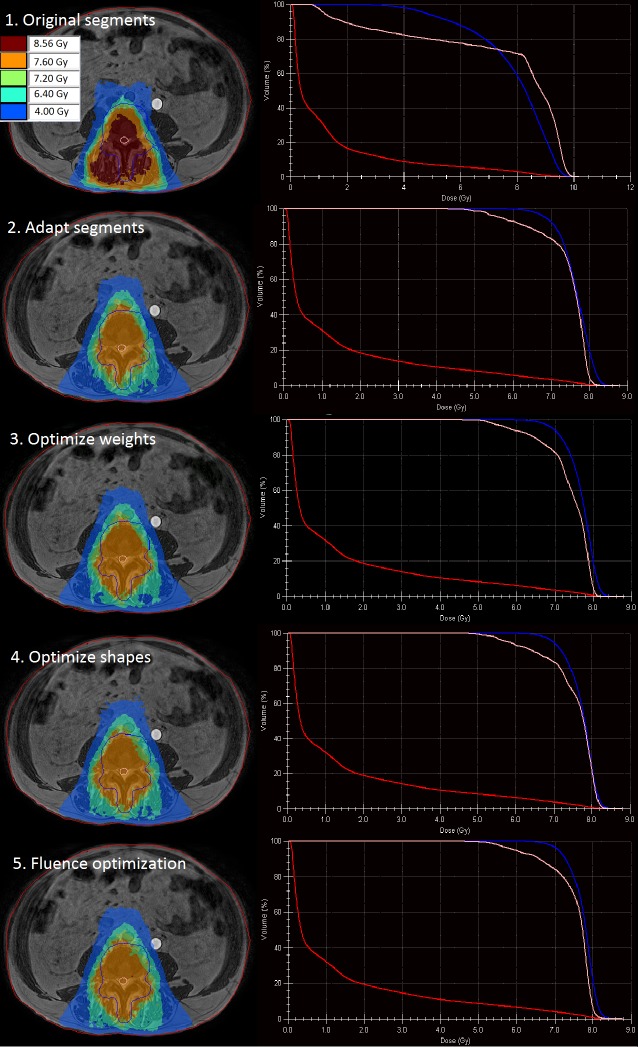
Dose distribution of the adapted plan using each of the five available plan adaptation methods (left) with the corresponding dose volume histograms (right). Visible are the planning target volume (PTV, blue), spinal cord (pink), and body (red).

Our results show large dosimetric variations between the used different plan adaptation methods (Figure 3, Table 2). Three of the plan adaptation methods, methods one, two, and three, resulted in insufficient target coverage, 75.4%, 86.6%, and 89.0%, respectively.

**Table 2:** Dosimetric outcomes of the five plan adaptation methods and full online replanning. Underdosage of the planning target volume (PTV) and violations of organ at risk (OAR) dose (Table 1) are denoted with an asterisk. PTV: planned target volume

DVH value	Pre-treatment	1. Original segments	2. Adapt segments	3. Optimize weights	4. Optimize shapes	5. Fluence optimization	Full replanning
PTV V_7.2Gy_ [%]	91.6	75.4*	86.6*	89.0*	90.0	92.5	97.1
PTV D_98%_ [Gy]	6.8	4.0*	6.5	6.7	6.7	6.8	7.1
PTV D_0.1cc_ [Gy]	8.7	9.9*	8.5	8.5	8.5	8.5	8.4
Spina cord D_0.1cc _[Gy]	8.2	9.9*	8.3	8.2	8.4	8.3	8.3
Body D_0.1cc_ [Gy]	8.1	9.5*	8.2	8.2	8.2	8.2	8.3
Timing [s]	n/a	10	11	13	30	32	223

The first method, in which original segments are kept and only the isocenter is modified, is expected to underperform in situations in which the patient is not exactly aligned as in the pre-treatment plan, which was the case for this patient. Using this method, no real adaptation is performed, as this is a simple recalculation of the dose for the patient in the new position. Because the plan segments were not modified, they were no longer correctly aligned with the target volume, resulting in an unfavorable dose distribution with high values to the spinal cord and the remainder of the body, D_0.1cc_ of 9.9Gy and 9.5Gy, respectively.

The second method, in which too the segments are modified based on the isocenter shift, showed a more favorable dose distribution. Dose to the OARs meets dose criteria; however, target coverage is insufficient with a planned target volume (PTV) V_7.2Gy_ of 86.6%. The results from methods one and two also indicate that using the original plan, even after adapting the segments based on the isocenter shift, is not a feasible option for this case. For this specific case, a better target coverage (PTV V_7.2Gy_ = 89.0%) was obtained using the third method in which the optimization of the segment weights is performed. The segment adaptation used in methods two and three is comparable to the situation of performing a couch shift. Because the shape of the segments is not modified to match the shape of the target, these methods are expected to perform less with target deformations.

Using the fourth method for plan adaptation, both the shapes and weights are optimized after adapting the segments. This method makes use of the segment aperture morphing (SAM) method [5] and adjusts the weight of the segments. For our case, this results in sufficient target coverage with a PTV V_7.2Gy_ of 90.0% and the dose to the OARs not violating any dose criteria. With the fifth method, fluence optimization, in which the segments are first removed, target coverage is further increased to a V_7.2Gy_ of 92.5%. Both methods take the shape of the target volume into account and are, therefore, expected to be most appropriate for plan adaptation in cases where the deformation of the target is likely.

**Figure 4:**
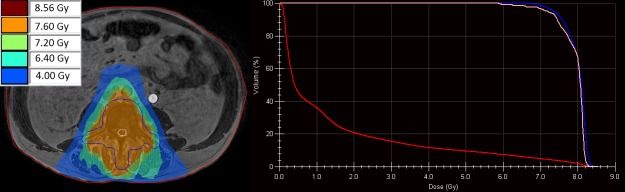
Dose distribution of the full online replanned plan (left) with the corresponding dose volume histogram (right). Visible are the planning target volume (PTV, blue), spinal cord (pink), and body (red).

By performing full online replanning (Figure 4), in which we create a new plan without segment or fluence information from a pre-treatment plan, the best target coverage is achieved (V_7.2Gy_ = 97.1%) while the dose to the OAR remains as low as with the available plan adaptation methods. Timings were measured using research hardware and range between 10 and 223 seconds. The investigated plan adaptation methods and full online replanning are expected to perform 1.5 to two times faster on clinical hardware.

## Discussion

These results show that multiples of the available plan adaptation methods generate an adapted treatment plan that meets all dose criteria and can successfully be used for patients with lumbar spine bone metastases in an online MR-guided workflow. Full online replanning, in which a complete new plan is generated on the daily anatomy without taking pre-treatment data into account, is the preferred method, as it optimally takes daily anatomy information into account. This case study also shows that full online replanning delivers the most favorable dose-volume histogram (DVH) parameters compared to the other plan adaptation methods. Although the dosimetric benefits are evident, further research must be performed to determine the clinical relevance.

For this particular case, it was possible to generate a full online replanned treatment plan in a time-frame suitable for online treatment on the 1.5T MR-linac. For larger target volumes or more complex types of treatment plans, full online replanning will take more time and may no longer be a feasible option, taking the total treatment time into account as well, so one of the alternative adaptation strategies has to be used.

Because our target volume is defined as the entire vertebral body, no non-rigid inter-fraction motion of the target had occurred between pre-treatment and the daily patient anatomy. Consequently, these results may not be indicative of soft-tissue targets in which daily organ deformations and changes in the shape and size of the target volume occur because not all available plan adaptation methods optimize the shape of the segments. These differences in inter-fraction motion, shape, and volume between different targets could potentially influence the most appropriate plan adaptation method. It is recommended to perform experiments for each specific type of radiotherapy treatment to determine the most appropriate method for plan adaptation.

## Conclusions

Our retrospective case report shows that for this case, multiple plan adaptation methods available at the 1.5T MR-linac are feasible to create clinically acceptable treatment plans in a timely manner suitable for online treatment. Full online replanning, in which a completely new plan is generated on the daily MRI with the current anatomy, yields the most beneficial DVH parameters.
